# Subclinical primary aldosteronism

**DOI:** 10.20945/2359-4292-2025-0400

**Published:** 2025-11-24

**Authors:** Stéfanie Parisien-La Salle, Jenifer M. Brown, Cheng Hsuan Tsai, Gregory L. Hundemer, Anand Vaidya

**Affiliations:** 1 Center for Adrenal Disorders, Division of Endocrinology, Diabetes, and Hypertension, Brigham and Women’s Hospital and Harvard Medical School, Boston, MA, United States; 2 Division of Endocrinology, Department of Medicine, Centre Hospitalier de l’Université de Montréal, Université de Montréal, Montréal, QC, Canada; 3 Division of Cardiovascular Medicine, Brigham and Women’s Hospital and Harvard Medical School, Boston, MA, United States; 4 Division of Cardiology, Department of Internal Medicine, National Taiwan University Hospital and National Taiwan University College of Medicine, Taipei, Taiwan; 5 Primary Aldosteronism Center at National Taiwan University Hospital, Taipei, Taiwan; 6 Department of Medicine, Division of Nephrology, University of Ottawa, Ottawa, ON, Canada

**Keywords:** Aldosterone, primary aldosteronism, subclinical primary aldosteronism, renin, adrenal

## Abstract

Primary aldosteronism (PA) is a major contributor to hypertension and cardiovascular disease. Subclinical PA, a preclinical or early manifestation of PA, has been identified in individuals with normal blood pressure and in those with mild hypertension without signs or symptoms of overt PA, using evidence from histopathology, hormonal biochemistry, proteomic, and genetic studies. Longitudinal studies have shown that subclinical PA increases the risk for developing incident hypertension, adverse cardiovascular remodeling, major adverse cardiovascular events, and chronic kidney disease. Given the clinical relevance of subclinical PA on the pathogenesis of cardiorenal disease, future studies should focus on methods to enhance early detection and assessment of the impact of early aldosterone-directed interventions for preventing adverse clinical outcomes.

## INTRODUCTION

Hypertension affects over 1.3 billion people worldwide and is the leading modifiable risk factor for both cardiovascular morbidity and mortality (^1^-^4^). Renin-independent, and relatively non-suppressible, aldosterone production, the underlying pathophysiology that characterizes primary aldosteronism (PA), can be found in over 25% of individuals with hypertension, translating to > 300 million individuals with underlying PA pathophysiology (^5^). This pathophysiologic aldosterone production results in inappropriate mineralocorticoid receptor (MR) activation, which contributes to cardiovascular disease through mechanisms such as oxidative stress, inflammation, and fibrosis (^6^). Compared to essential hypertension, PA carries a disproportionately higher risk of cardiovascular complications, independent of blood pressure (^7^). Identification of PA pathophysiology is crucial, as aldosterone-directed therapy can reduce the risk for cardiovascular disease (^8^,^9^). For this reason, modern clinical practice guidelines now recommend screening for PA for all individuals with hypertension (^3^,^10^).

The recent paradigm shift viewing PA as a spectrum rather than a dichotomous disease has enabled more research into its earliest manifestations, referred to as subclinical PA (^5^,^11^-^16^). Subclinical PA refers to the identification of biochemical renin-independent aldosterone production in individuals with normal blood pressure and those with mild hypertension without signs or symptoms of overt PA. Herein, we comprehensively review the evidence on the underlying pathogenesis and pathophysiology of subclinical PA and its relevance to adverse cardiovascular outcomes.

## THE PATHOGENESIS AND BIOCHEMICAL SIGNATURE OF SUBCLINICAL PA

Subclinical PA has been characterized using multiple modalities, including histopathology, steroid and hormonal biochemistry, proteomics, and aberrant receptor expression.

### Histopathology

The development of immunohistochemistry for aldosterone synthase has been an important breakthrough in understanding the cellular origins of PA (^17^,^18^). Identification of what were initially termed aldosterone producing cell clusters (APCCs) through CYP11B2 staining helped redefine the pathology of PA from the dichotomous designation of unilateral adenoma vs bilateral hyperplasia towards a more nuanced understanding of PA (^19^,^20^). These APCCs, now referred to as aldosterone producing micronodules (APMs) were shown to frequently harbor pathogenic somatic driver variants for PA, elevated CYP11B2 expression, and hypothesized to be precursor lesions to aldosterone producing adenomas (APAs) (^21^-^24^). APMs harboring pathogenic somatic mutations can be detected in morphologically normal adrenal glands from individuals with normal blood pressure (^24^-^26^), and accumulate with age (^26^-^28^). These findings suggest that dysregulated expression of CYP11B2 is common, precedes the onset of overt PA, may characterize the subclinical phases of the disease, and could explain a large proportion of agerelated hypertension.

### Hormonal biochemistry

Many studies have demonstrated a biochemical spectrum of renin-independent aldosterone production in normotensive people (^5^,^11^,^12^,^14^-^16^,^29^). As aldosterone and renin levels exhibit significant variability (^30^), this continuum has been demonstrated using postural and dietary maneuvers to evaluate the regulation of the renin-angiotensin-aldosterone system (RAAS) system, including: sodium restriction and upright posture to stimulate plasma renin and angiotensin II (AngII) (^14^), seated sodium loading test (SST) to suppress aldosterone production (^16^), oral sodium loading test to suppress aldosterone production (^11^,^12^), and supine posture following oral sodium loading to suppress aldosterone production (^15^).

Using these carefully conducted phenotyping maneuvers, a continuum of renin-independent aldosterone production has been described in individuals with normal blood pressure that exhibits parallel responsiveness to both AngII and adrenocorticotropic hormone (ACTH) (^5^,^12^,^15^,^16^), akin to phenomena that have been observed in patients with overt PA (^31^-^33^). The altered sensitivity, or increased responsiveness, to these ligands may be driven by upregulation or sensitization of MC2-R (ACTH receptors) and AT1-R (type 1 angiotensin II receptors) in aldosterone-producing regions, with expression levels differing by genotype (^34^-^37^).

Another biochemical feature associated with subclinical PA is natriuretic peptide (NP) insufficiency (^15^). This finding was first observed in a populationbased epidemiological study demonstrating an inverse relationship between aldosterone and NP levels in normotensive people (^38^). Aldosterone and NPs are reciprocal hormonal systems that regulate each other via intravascular volume but also via direct regulatory effects (^39^-^42^). In subclinical PA, aldosterone production results in NP insufficiency, which may then contribute to impaired natriuresis, vasodilation, and the inability to suppress aldosterone production, leading to the synergistic development of hypertension, fibrosis, and inflammation.

A recent study showed that in normotensive people, the magnitude of subclinical PA correlated with 18-hybrid steroid production (^12^). 18-hybrid steroids are well-established biomarkers of PA pathophysiology, reflecting dysregulated CYP11B2 activity and its co-expression with CYP17A1 (^43^,^44^). The demonstration that normotensive people exhibit a spectrum of renin-independent aldosterone production that correlates with 18-hydroxycortisol and 18-oxocortisol production supports the notion that there is a pathologic continuum of PA detectable among normotensive people.

### Proteomics

The plasma proteome of PA has been previously described when compared to people with hypertension revealing dysregulated pathways related to inflammation, including neutrophil degranulation, phagocytosis, regulation of reactive oxygen species, and oxidative stress-induced apoptosis (^45^). These pathways were reinforced by another large-scale proteomic study which characterized the proteomic evolution of subclinical PA to overt PA (^46^). The proteomic signature of PA originated in normotensive people and was characterized by pathways implicated in cardiovascular disease pathogenesis including inflammation, vascular remodeling and redox imbalance (^46^). Within this study, Norrin or *NDP*, a Wnt/β-catenin ligand, showed robust, aldosterone dose-dependent trends across various physiological maneuvers, suggesting its potential role as a novel regulator in the pathogenesis of PA (^46^).

### Aberrant receptor expression and other mechanisms

Aldosterone production has been shown to be regulated by eutopic and ectopic receptors in adrenal cortical tissue: AT1-R and MC2-R as described above, but also receptors for vasopressin, serotonin, GIP, leptin, mast cells, LH/HCG and neuropeptide substance P (^47^-^52^). The role of these ectopic receptors in the pathogenesis of subclinical PA remains to be studied directly.

Many other hypotheses have also been proposed for the origins of PA, including a two-hit model, where an initial germline pathogenic variant drives tumoral proliferation and a somatic pathogenic variant in PA susceptibility genes promotes aldosterone production (^53^,^54^).

Finally, the germline SNPs identified in genomewide association studies of PA patients highlight a genetic susceptibility that predates clinical diagnosis (^55^-^57^). These genes include *CASZ1, RXFP2, LSP1, NDP, WNT2B, CYP11B1/2, TBX3, HOTTIP* and *ADGRB3* (^55^-^57^). Notably, the involvement of *NDP* in PA pathogenesis was subsequently validated in a study including normotensive and hypertensive people where SNPs in *NDP* were associated with dosedependent increases in aldosterone production (^46^).

## SUBCLINICAL PA AND RISK FOR INCIDENT HYPERTENSION

Subclinical PA has been associated with an increased risk for incident hypertension in longitudinal studies (^13^,^58^-^61^). A large prospective study from Canada found that a higher aldosterone-to-renin ratio (ARR) was associated with a greater risk of incident hypertension (adjusted OR, 1.29 [95% CI, 1.03-1.62]) (^58^). Similarly, a study by Markou and cols. found that normotensive individuals with elevated postfludrocortisone-dexamethasone suppression test aldosterone levels and ARR had a significantly higher risk of developing hypertension over five years (^59^). Supporting this, Brown and cols. identified that higher aldosterone concentrations in the context of a low renin phenotype were associated with an increased risk for incident hypertension (^13^). A longitudinal analysis from adolescence (age 17) to adulthood (age 27) demonstrated a positive correlation between ARR at 17 and blood pressure at 27, suggesting that elevated adolescent ARR may predict future hypertension (^62^).

## SUBCLINICAL PA AND RISK FOR INCIDENT MAJOR ADVERSE CARDIOVASCULAR OUTCOMES

Many studies have shown that the magnitude of the subclinical PA biochemical phenotype predicts the risk for incident cardiovascular disease (^14^,^58^,^63^-^66^).

### Atherosclerosis, cardiac structure and function

A large study using data from the Multi-Ethnic Study of Atherosclerosis (MESA), which included adults free of cardiovascular disease at baseline and not taking blood pressure medications, found that higher aldosterone levels were associated with a higher coronary artery calcium, particularly when renin was low (^65^). It should be noted that the overall mean blood pressure in the cohort was 118.6 ± 18.5/69.4 ± 9.5 with approximately 85% of the participants considered to be normotensive (^65^). A retrospective analysis from a subgroup of normotensive participants from the longitudinal Atherosclerosis Risk in Communities (ARIC) study revealed that a low renin phenotype was associated with greater left ventricular (LV) mass index, LV end-diastolic and end-systolic volumes, and left atrial volume index (^64^).

The deleterious impact of elevated aldosterone and ARR on cardiac structure was also demonstrated in individuals with familial hyperaldosteronism type I (FH-I) compared with matched controls, both groups having normal blood pressure (^66^). FH-I patients showed greater left ventricular wall thickness and impaired diastolic function, highlighting that aldosterone excess promotes adverse cardiac remodeling even in the absence of hypertension (^66^). Finally, a longitudinal cohort study from the CARTaGENE cohort demonstrated that the magnitude of subclinical PA was associated with greater arterial stiffness, evidenced by elevated central blood pressure and pulse wave velocity, as well as adverse cardiac remodeling, such as increased left atrial volume index, LV mass index, remodeling index and LV hypertrophy as measured by cardiac MRI (^58^). A sensitivity analysis excluding participants taking antihypertensive medication yielded similar results to the primary analyses (^58^).

### Clinical outcomes

The CARTaGENE cohort of mostly normotensive people also yielded data linking subclinical PA magnitude to major adverse cardiovascular events (MACE), described as a composite of myocardial infarction, stroke (ischemic or hemorrhagic), hospitalization for heart failure, and cardiovascular death (^63^). Both the low renin phenotype (adjusted hazard ratio, 2.22 [95% CI, 1.02-4.76]) and higher ARR (adjusted hazard ratio, 2.43 [95% CI, 1.15-5.12]) were associated with a higher risk for MACE (^63^).

In the MESA cohort described above, aldosterone was associated with the increased risk of all-cause mortality when plasma renin activity was low over a median follow-up of 12.5 years (^65^).

## SUBCLINICAL PA AND CHRONIC KIDNEY DISEASE (CKD)

A large study of more than 1900 healthy communitydwelling individuals found that both aldosterone and ARR were inversely associated with glomerular filtration rate, independent of multiple confounders including blood pressure, diabetes status and weight. Importantly, higher aldosterone was significantly associated with an increased risk of chronic kidney disease (^67^). In a study by Terata and cols., conducted in Japanese individuals without CKD and not on antihypertensive medication with an observation period of over 9 years, individuals who developed CKD had significantly lower baseline plasma renin activity and higher baseline ARR level (^68^).

## CONCLUSION

**Figure 1:**
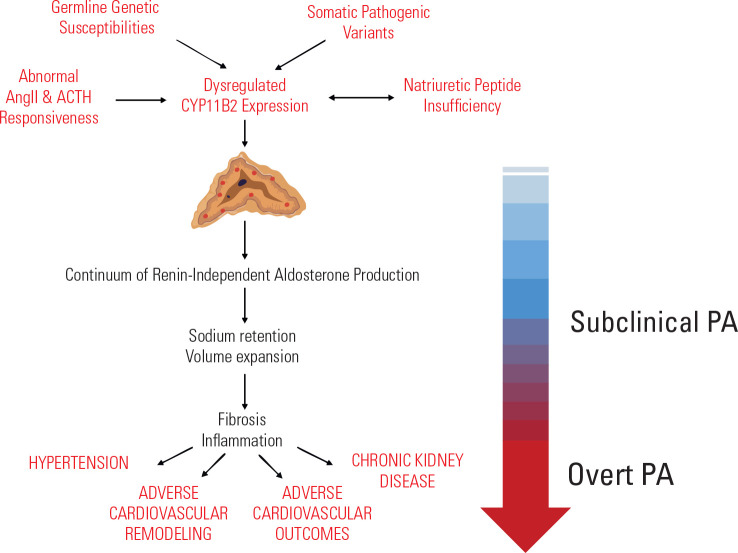
The pathophysiology of subclinical primary aldosteronism.

Subclinical PA is a biochemical phenotype characterized by renin-independent, and relatively non-suppressible, aldosterone production that originates in normotensive people and precedes the development of hypertension. Subclinical PA is associated with higher risks for incident hypertension, cardiovascular disease, and CKD (**Figure 1**). The importance of recognizing this phenotype lies in the global burden of hypertension and the opportunity to identify mechanisms that allow targeted therapy with widely available mineralocorticoid receptor antagonists, and that may also be a potential target for emerging treatments such as aldosterone synthase inhibitors (^69^). However, more practical approaches are needed to identify pathophysiological aldosterone production without relying on complex physiological testing (^70^). Future efforts should focus on earlier and more pragmatic identification of subclinical PA pathophysiology, and the impact of early implementation of aldosterone-directed interventions on clinical outcomes.

Subclinical PA is characterized by a continuum of non-suppressible, renin-independent aldosterone production, in normotensive or mildly hypertensive people without overt PA. The interplay of germline genetic susceptibilities, somatic pathogenic variants, natriuretic peptide insufficiency, and abnormal responsiveness to AngII and ACTH lead to dysregulated CYP11B2 expression and aldosterone production. Pathophysiologic aldosterone production causes sodium retention, volume expansion, fibrosis and inflammation, ultimately leading to hypertension, adverse cardiovascular remodeling and clinical outcomes.

## Data Availability

datasets related to this article will be available upon request to the corresponding author.
